# Effect of total intravenous opioid-free anesthesia on quality of recovery following gynecological laparoscopy: protocol for a multicenter, randomized, double-blind, controlled trial

**DOI:** 10.3389/fmed.2026.1747328

**Published:** 2026-02-09

**Authors:** Ting-ting Wu, Ke Peng, Yu-hao Fu, Yan Sun, Yi-shan Lei, Hong Liu, Xi-sheng Shan, Fu-hai Ji

**Affiliations:** 1 Department of Anesthesiology, First Affiliated Hospital of Soochow University, Suzhou, Jiangsu, China; 2 Institute of Anesthesiology, Soochow University, Suzhou, Jiangsu, China; 3 Department of Anesthesiology and Pain Medicine, University of California Davis Health, Sacramento, CA, United States

**Keywords:** gynecological laparoscopic surgery, opioid-based anesthesia, opioid-free anesthesia, postoperative nausea and vomiting, quality of recovery

## Abstract

**Background:**

Whether opioid-free anesthesia (OFA) improves postoperative quality of recovery remains uncertain. This study aims to compare the effects of an intraoperative OFA protocol with traditional intraoperative opioid-based anesthesia (OBA) on recovery quality in patients undergoing gynecological laparoscopic surgery.

**Methods:**

This multicenter, randomized, double-blind, controlled trial will include 300 adult women scheduled for elective gynecological laparoscopic surgery at five tertiary hospitals in China. Patients will be randomized in a 1:1 ratio to either the OFA group (dexmedetomidine, esketamine, and lidocaine) or OBA group (sufentanil), stratified by study center. Following anesthesia induction, all patients will receive bilateral transversus abdominis plane blocks and propofol-based total intravenous anesthesia. The primary outcome is postoperative quality of recovery at 24 h, assessed using the Quality of Recovery−15 (QoR-15) questionnaire. Secondary outcomes include incidence of postoperative nausea and vomiting, QoR-15 scores at 48 and 72 h, numeric rating scale pain scores at rest and on coughing, cumulative opioid consumption, health-related quality of life and incidence of chronic pain at 3 months. Adverse events include hypotension, bradycardia, hypertension, tachycardia, oversedation, desaturation, dizziness, headache, ileus, hyperalgesia, psychiatric related side effects (hallucinations, agitation, nightmares, or delirium) occurring intraoperatively or during hospitalization. The primary analysis will be conducted according to the modified intention-to-treat principle.

**Discussion:**

We hypothesize that an intraoperative intravenous OFA regimen will enhance recovery quality compared with a traditional intraoperative OBA regimen in women undergoing gynecological laparoscopic surgery. The findings are expected to inform evidence-based optimization of anesthetic strategies in this surgical population.

**Trial registration number:**

https://www.chictr.org.cn, identifier (ChiCTR2500106392).

## Introduction

Minimally invasive surgery, particularly gynecological laparoscopy, has become the standard of care for many gynecological conditions, offering reduced surgical trauma and faster recovery compared with open procedures (1). Despite these advantages, gynecological laparoscopy is still associated with several complications, most notably postoperative nausea and vomiting (PONV), which may increase the risk of postoperative hemorrhage, delay recovery, and prolong hospitalization, thereby significantly impairing overall recovery quality (2–6).

Opioid-free anesthesia (OFA) is a multimodal strategy that avoids intraoperative opioids by combining the use of α2-adrenergic agonists, N-methyl-D-aspartate (NMDA) antagonists, magnesium sulfate, and local anesthetics to reduce opioid-related complications, such as PONV, gastrointestinal dysfunction, and respiratory depression (7–9). Small studies in thoracic, thyroid, and hip surgery have suggested that OFA may reduce these adverse events and improve analgesia (10–12). Choi et al. reported that an OFA regimen with dexmedetomidine and lidocaine significantly improved recovery quality in patients undergoing gynecological laparoscopic surgery (13), and Chen et al. found that OFA with esketamine and dexmedetomidine was non-inferior to traditional opioid-based anesthesia (OBA) in terms of analgesia and intraoperative hemodynamics, while reducing PONV and improving postoperative sleep quality, albeit with prolonged emergence (14). Conversely, another randomized trial found only a reduction in early PONV by OFA and similar pain control and overall recovery quality compared to OBA (15). These conflicting findings derived from small sample and heterogeneous studies highlight the need for a multicenter trial evaluating OFA in gynecological laparoscopy.

This multicenter, randomized controlled trial will test the hypothesis that an intraoperative intravenous OFA regimen comprising dexmedetomidine, esketamine, lidocaine, and propofol would improve the quality of recovery compared with a traditional intraoperative OBA regimen using sufentanil and propofol in women undergoing gynecological laparoscopic surgery.

## Methods

### Ethics and registration

The protocol was approved by the Clinical Research Ethical Committee of the leading center (First Affiliated Hospital of Soochow University; Approval No. 2024–554) and all participating centers (Nanjing Maternity and Child Health Care Hospital, Hebei General Hospital, Yijishan Hospital of Wannan Medical College, and Shanxi Bethune Hospital). This trial was registered on the Chinese Clinical Trial Registry (identifier: ChiCTR2500106392) on 23 July 2025 prior to patient enrollment. Written informed consent will be obtained from all participants.

### Study design and participants

**Figure 1:**
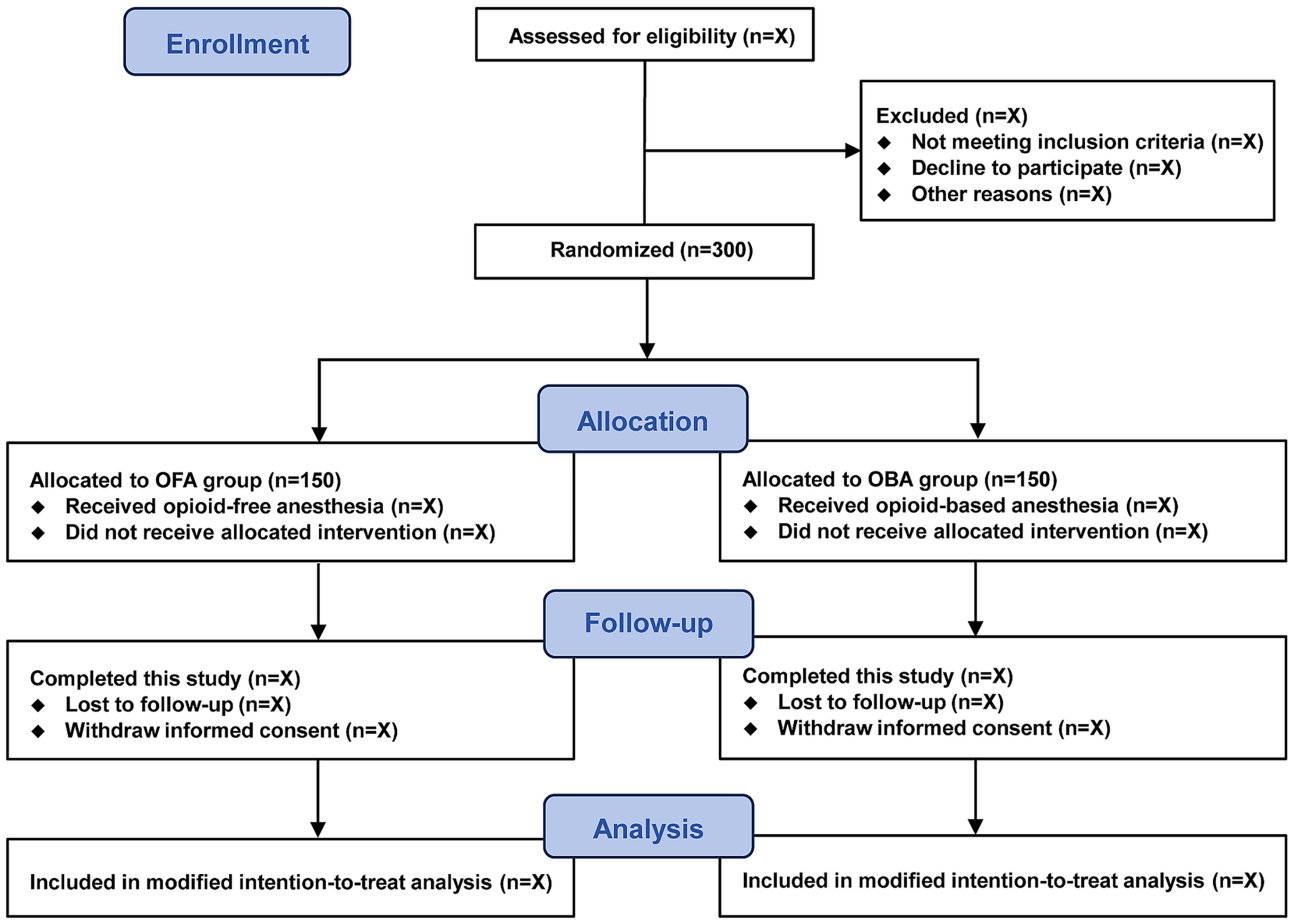
Study flowchart.

This investigator-initiated, multicenter, double-blind, randomized controlled trial is being conducted at five tertiary hospitals in China. This trial commenced in August 2025 and is anticipated to conclude in August 2027. A total of 300 eligible patients undergoing elective gynecological laparoscopic surgery will be included. The study flow diagram is presented in Figure 1. In accordance with the SPIRIT statement (Supplementary file 1), the schedule of patient recruitment, study interventions, and outcome assessments is summarized in Table 1.

**Table 1:** Schedule of patient enrollment, study interventions, and outcome measurements.

Time point	Study period								
	Enrollment	Allocation	Post-allocation						Close-out
	-1 to 0 day before surgery	2 h before surgery	During surgery	PACU	24 h after surgery	48 h after surgery	72 h after surgery	Hospital discharge	3 months after surgery
Enrollment									
Eligibility screening	╳								
Written informed consent	╳								
Demographic data	╳								
Baseline characteristics	╳								
Randomization		╳							
Allocation		╳							
Interventions									
Opioid-free anesthesia			╳						
Opioid-based anesthesia			╳						
Measurements									
QoR-15 scores					╳	╳	╳		
Extubation time			╳	╳					
Length of PACU stay				╳					
Incidence of PONV				╳	╳	╳	╳	╳	
NRS pain scores					╳	╳			
Total opioid consumption					╳	╳	╳	╳	
Patient satisfaction					╳				
Postoperative hospital stay								╳	
Health-related quality of life at 3 months									╳
Chronic pain at 3 months									╳
Adverse events^a^			╳	╳	╳	╳	╳	╳	

According to SPIRIT statement of defining standard protocol items for clinical trials. PACU, post-anesthesia care unit; PONV, postoperative nausea and vomiting; QoR-15, Quality of Recovery-15.

a Including hypotension, bradycardia, hypertension, tachycardia, oversedation, desaturation, dizziness, headache, ileus, hyperalgesia, psychiatric related side effects (Hallucinations, agitation, nightmares, or delirium).

Eligible participants included: (1) female patients, (2) aged 18–65 years, (3) American Society of Anesthesiologists (ASA) physical status I–III, and (4) scheduled for elective gynecological laparoscopic surgery under endotracheal general anesthesia.

Exclusion criteria were as follows (1) pregnancy, planned pregnancy, or breastfeeding, (2) body mass index (BMI) < 18 kg/m^2^ or > 35 kg/m^2^, (3) chronic pain lasting > 3 months or current use of sedatives or analgesics, (4) uncontrolled psychiatric disorders (e.g., anxiety, depression, schizophrenia), (5) uncontrolled hypertension (blood pressure > 160/100 mmHg despite treatment), (6) sick sinus syndrome, severe bradycardia (< 50 beats/min), or heart block, (7) left ventricular ejection fraction < 40%, (8) unstable coronary artery disease, (9) gastric ulcer or active gastrointestinal bleeding, (10) hepatic or renal dysfunction, (10) history of porphyria, epilepsy, Parkinson’s disease, or Alzheimer’s disease, (11) known allergy to any study medication, or (12) inability to communicate effectively.

### Randomization and blindness

An independent statistician with no other study involvement will generate the randomization sequence using SAS statistical software (version 9.4, SAS Institute Inc.). Participants will be randomized in a 1:1 ratio with blocks of 4, stratified by study center. The allocation sequence will be concealed in sequentially numbered, opaque, sealed envelopes, stored in a locked cabinet until enrollment. An independent researcher, responsible for participant enrollment, will open the assigned envelope, prepare the study medications, and label them with a unique medication number and patient number. All study solutions (dexmedetomidine, esketamine, lidocaine, sufentanil, and normal saline placebo) are colorless and transparent, which are visually indistinguishable. Patients, anesthesiologists, surgeons, and outcome assessors will remain blinded until completion of the final analyses.

### Study interventions

The study intervention procedures are summarized in Table 2. All study medications were dispensed in syringes labeled only with a study-drug number, with identical syringe appearance and fill volume across groups (e.g., ‘Study drug 1’ denoted dexmedetomidine or its matched placebo). During anesthesia induction, patients in the OFA group will receive dexmedetomidine (0.6 μg/kg over 10 min, followed by 0.2–0.7 μg/kg/h; Study drug 1), propofol 2.0–2.5 mg/kg, esketamine 0.3 mg/kg (Study drug 2), and lidocaine 1 mg/kg (Study drug 3). Patients in the OBA group will receive a normal saline infusion (Study drug 1) administered at the same rate as dexmedetomidine, propofol 2.0–2.5 mg/kg, sufentanil 0.3 μg/kg (Study drug 2), and volume-matched saline in place of lidocaine (Study drug 3). For anesthesia maintenance, both groups will undergo total intravenous anesthesia (TIVA) with propofol at 50–150 μg/kg/min. Intraoperatively, the OFA group will receive esketamine 0.1–0.2 mg/kg boluses (Study drug 4) as required, whereas the OBA group will receive sufentanil 0.1–0.2 μg/kg boluses (Study drug 4) as clinically indicated.

**Table 2:** Details of study interventions.

Study drug	Usage	OFA group (*n* = 150)	OBA group (*n* = 150)
Anesthesia induction			
Study drug 1	Infusion	Dexmedetomidine 0.6 μg/kg + 0.2─0.7 μg/kg/h, 200 μg/50 mL	Normal saline, 50 ml
Propofol	Bolus	Propofol 2.0–2.5 mg/kg	Propofol 2.0–2.5 mg/kg
Study drug 2	Bolus	Esketamine 0.3 mg/kg, 50 mg/20 ml	Sufentanil 0.3 μg/kg, 50 μg/20 ml
Study drug 3	Bolus	Lidocaine 1 mg/kg, 100 mg/10 ml	Normal saline, 10 ml
Anesthesia maintenance			
Propofol	Infusion	Propofol 50–150 μg/kg/min	Propofol 50–150 μg/kg/min
Study drug 4	Bolus	Esketamine 0.1–0.2 mg/kg boluses, 50 mg/20 ml	Sufentanil 0.1–0.2 μg/kg boluses, 50 μg/20 mL

OFA, opioid-free anesthesia; OBA, opioid-based anesthesia.

### Perioperative management

All patients will fast for 8 h (clear fluids permitted until 2 h before surgery). No premedication will be administered. Baseline blood pressure will be measured in the preoperative waiting area. In the operating room, standard monitoring will include electrocardiography, non-invasive cuff blood pressure, and pulse oximetry (SpO2), and nasopharyngeal temperature. Depth of anesthetic will be monitored using the Bispectral Index (BIS, Aspect Medical Systems, Newton, MA).

After induction with the allocated regimen (Table 2), tracheal intubation will be facilitated with intravenous rocuronium 0.6 mg/kg. Mechanical ventilation will be initiated with a tidal volume of 8–10 mL/kg (predicted body weight), respiratory rate of 12–15 breaths/min, fraction of inspired oxygen of 60%, and positive end-expiratory pressure of 3–5 cmH_2_O. End-tidal carbon dioxide will be maintained at 35–45 mmHg. Following induction, bilateral ultrasound-guided transversus abdominis plane (TAP) blocks will be performed using 20 mL of 0.25% ropivacaine per side. Anesthesia depth will be maintained within a BIS range of 40–60 by titrating the propofol infusion. Supplemental analgesics and neuromuscular blockers will be administered based on surgical stimulation and hemodynamic responses. Patients will be covered with a warming blanket to maintain nasopharyngeal temperature at 36–37 °C. Lactated Ringer’s solution will be the default intravenous fluid. At the end of surgery, patients will be transferred to the post-anesthesia care unit (PACU) and subsequently to the surgical ward. A modified Aldrete Score ≥ 9 will be required for PACU discharge.

Hemodynamic events will be managed according to predefined protocols. Hypotension, defined as mean blood pressure [MBP] decrease of > 30% from baseline or MBP < 65 mmHg, will be managed with intravenous ephedrine (6–10 mg) or phenylephrine (50–100 μg). Bradycardia, defined as HR < 50 beats/min, will be treated with intravenous atropine (0.3–0.5 mg) or ephedrine (6–10 mg) if coexisting hypotension. Hypertension, defined as MBP increase of > 30% from baseline will be controlled with intravenous urapidil 5–10 mg. Tachycardia, defined as HR > 100 beats/min, will be controlled with intravenous esmolol 10–20 mg. Desaturation, defined as peripheral SpO2 < 90% after extubation, will be managed with supplemental oxygen at 5–10 L/min via the nasal catheter. In the event of upper airway obstruction, appropriate interventions will be undertaken, including jaw thrust, chin lift, placement of an oropharyngeal airway, or ventilation using a facemask or laryngeal mask, as indicated.

PONV prophylaxis include intravenous dexamethasone 5 mg after induction and ondansetron 4 mg at the end of surgery. Flurbiprofen axetil 50 mg will also be administered intravenously. Postoperative multimodal analgesia will consist of intravenous flurbiprofen axetil 50 mg once daily on postoperative days 1 and 2, and patient-controlled intravenous analgesia (PCIA). The PCIA will be prepared with sufentanil 100 μg diluted to 100 mL with normal saline, delivered with no background infusion, bolus 2 mL and a 10 min lockout interval. PCIA will be initiated in the PACU. Patients will be instructed to self-administer sufentanil when the numeric rating scale (NRS, 0–10; 0 = no pain, 10 = worst imaginable pain) score is ≥ 4. If the pain remains uncontrolled, rescue morphine 2–5 mg or other opioids (tramadol, butorphanol, dezocine) will be provided. PCIA will be discontinued 48 h postoperatively. For rescue antiemetic therapy, 5-HT₃ receptor antagonists (ondansetron, granisetron, or tropisetron) will be administered as required.

### Data collection

Demographic data, baseline characteristics, and study outcomes will be collected by trained independent investigators who are blinded to the group assignment. The Quality of Recovery-15 (QoR-15) questionnaire will be completed at 24, 48, 72 h postoperatively. If a patient is discharged before the 72-h assessment, the evaluations will be conducted by telephone.

All study data will be documented in electronic case report forms (eCRFs) by trained research personnel. On completion of data entry, the eCRFs will be compiled into a de-identified electronic trial database, which will be locked. The secured dataset will subsequently be transferred to an independent statistician for analysis in accordance with the prespecified statistical analysis plan.

An independent Data Monitoring Committee (DMC) has been established, comprising a chair (an experienced anesthesiologist), a pharmacologist, a gynecologist and a statistician. The DMC will oversee the overall conduct of the trial, inspect data quality and resolve issues related to data collection or registration. In case of uncertainties or discrepancies, the DMC will convene to review the matter and determine a consensus resolution. Any adverse event related to the study interventions must be reported to the DMC using the designated “Adverse Event Form” within 24 h. Upon occurrence of a serious adverse event, such as an unanticipated deterioration in the patient’s perioperative clinical condition, the attending anesthesiologist could request unblinding the treatment allocation and adjust patient management as required.

### Study outcomes

The primary outcome of this study is quality of recovery at 24 h postoperatively, assessed using the QoR-15 questionnaire. The QoR-15 is a validated patient-reported outcome measure comprising 15 items, each scored on an 11-point Likert scale (0–10), yielding a total score ranging from 0 to 150 (0 = extremely poor recovery; 150 = excellent recovery).

Secondary outcomes include: (1) incidence of postoperative nausea and vomiting (PONV); (2) QoR-15 scores at 48 and 72 h postoperatively; (3) NRS pain scores (at rest and during coughing) in the PACU, and at 24 and 48 h postoperatively; (4) total opioid consumption at 0–24 and 24–48 h, converted to morphine milligram equivalents; (5) health-related quality of life at 3 months, evaluated using the EuroQol-Visual Analog Scale (EQ-VAS) (16); (6) incidence of chronic pain at 3 months postoperatively, defined as an NRS score ≥ 1.

Other outcomes include: (1) time to tracheal extubation; (2) length of PACU stay; (3) patient satisfaction at 24 h postoperatively, assessed using the NRS (0 = completely dissatisfied; 10 = fully satisfied); (4) length of postoperative hospital stay.

Safety outcomes will be evaluated separately and in aggregate for all patients, consisting of adverse events occurring intraoperatively or during hospitalization. Adverse events include hypotension, bradycardia, hypertension, tachycardia, oversedation, desaturation, dizziness, headache, ileus, hyperalgesia, psychiatric related side effects (Hallucinations, agitation, nightmares, or delirium). The definitions of adverse events are based on the Common Terminology Criteria for Adverse Events (CTCAE─Version 5.0; Supplementary file 2)

### Sample size

The minimal clinically important difference (MCID) for the QoR-15 score had been established as 6 points (17). Between January and March 2025, we conducted a prospective study involving two cohorts of 10 patients each who underwent gynecological surgery using either an OFA or OBA protocol. The overall 24-h QoR-15 score was 112 ± 15 (mean ± SD). Based on those preliminary data, assuming a two-sided significance level (*α*) of 0.05, a standard deviation of 15, and a statistical power (1-*β*) of 90%, the required sample size was calculated to be 133 patients per group. To account for an anticipated 10% dropout rate, we plan to enroll 300 patients (PASS version 15.0.5, PASS Institute Inc.).

### Statistical analysis

Continuous variables will be tested for normality using the Shapiro–Wilk test and presented as mean (SD) or median with interquartile range (IQR), with between-group comparisons using the *t*-test or Mann–Whitney *U* test as appropriate. Categorical variables will be reported as counts (%) and compared using the chi-squared or Fisher’s exact test.

Demographic and baseline characteristics will be summarized descriptively, without conducting between-group hypothesis testing. For study outcomes, the treatment effects between groups will be evaluated using odds ratios (OR) or mean differences (MDs), each with 95% confidence intervals (CIs). Multivariate logistic regression or generalized linear models will be applied to further analyze outcomes, adjusting for relevant baseline covariates (age, BMI, surgical procedure, number of ports, and surgical duration) and study center. Subgroup analyses of the primary outcome will be conducted to assess potential heterogeneity of treatment effects across predefined subgroups, including BMI (< 25 kg/m^2^ vs. ≥ 25 kg/m^2^), history of hypertension and diabetes mellitus (yes vs. no), menopause (yes vs. no), surgical procedure, number of ports (single vs. multiple), and study center (leading vs. participating), with interaction terms evaluated using logistic regression. For secondary outcomes, multiple testing will be adjusted using the Benjamini–Hochberg method to control the false discovery rate.

The primary analysis will follow the modified intention-to-treat principle, including all randomized patients who undergo surgery and have available data for the primary outcome. To assess the robustness of the primary findings, sensitivity analyses will be performed using additional analysis sets, including an intention-to-treat analysis and a per-protocol analysis. In the intention-to-treat analysis, missing data for the primary outcome will be handled using multiple imputation by chained equations, assuming data are missing at random. A per-protocol analysis will exclude patients with major protocol deviations, including conversion to open laparotomy, failure to adhere to the assigned analgesic protocol, and reoperation or additional surgical procedures within the first 24 postoperative hours. Secondary and other outcomes will be analyzed using observed data only. No interim analyses are planned. All statistical tests will be two-sided, with statistical significance defined as *p* < 0.05. Analyses will be conducted using R software (version 4.3.0; R Foundation for Statistical Computing).

## Discussion

This multicenter, randomized, double-blind controlled trial will enroll 300 eligible patients to evaluate the effects of intraoperative OFA versus intraoperative OBA on recovery quality following gynecological laparoscopic surgery. In addition, we will also evaluate nausea and vomiting, postoperative pain, opioid consumption, health-related quality of life, incidence of chronic pain, and postoperative adverse events and complications. The primary hypothesis is that a propofol-based intravenous OFA regimen improves postoperative recovery quality compared with traditional OBA. The trial will adhere to the Consolidated Standards of Reporting Trials guidelines (18).

Opioids have long been regarded as the mainstay of intraoperative analgesia; however, perioperative opioid use is associated with several adverse effects, including dizziness, sedation, delirium, PONV, hyperalgesia, respiratory depression, and constipation (9). Furthermore, opioid use has been shown to modulate the immune system, suppress antibody production, inhibit cellular immune responses, reduce natural killer cell and phagocytic activity, and perturb cytokine expression, all of which may adversely affect oncological outcomes (9, 19). In gynecological laparoscopic surgery, the laparoscopic approach, the specific gynecologic procedures, and female sex are independent risk factors for PONV. When combined with the frequent perioperative opioid administration, the incidence of PONV in this population can reach 80%, markedly compromising recovery quality (2–4). Accordingly, OFA may confer potential benefits for this high-risk population.

In this study, we examine whether the intraoperative OFA strategy with perioperative multimodal analgesia can enhance recovery quality after gynecological laparoscopy. In the OFA regimen, intraoperative antinociception is provided by esketamine, dexmedetomidine, and lidocaine, in combination with TAP blocks. Esketamine, the S (+) enantiomer of ketamine, acts primarily on NMDA receptors, exerting both anti-nociceptive and anesthetic effects (20). Dexmedetomidine, a highly selective α2-adrenergic receptor agonist, possesses sedative, analgesic, and anxiolytic properties (21). When administered together, esketamine and dexmedetomidine achieve a balanced sedative-analgesic profile, with the sedative and anxiolytic actions of dexmedetomidine mitigating the psychotropic adverse effects of esketamine (22). Intravenous lidocaine exerts systemic antinociceptive effects, reducing postoperative pain, opioid requirements, and the risk of hyperalgesia (23–25). The TAP blocks, a commonly adopted regional anesthesia technique, provide reliable somatic analgesia for laparoscopic surgery (26, 27). Furthermore, the enhanced recovery after surgery (ERAS) guidelines for gynecological surgery recommend TAP blocks as an effective component of perioperative multimodal analgesia (28).

Early OFA protocols primarily focused on analgesic efficacy and opioid-sparing effects. More recently, attention has shifted to postoperative quality of recovery, a core concept in perioperative medicine that encompasses patient-reported restoration of preoperative function and alleviation of adverse symptoms (29). A recent meta-analysis of 13 randomized controlled trials demonstrated that OFA enhances early postoperative quality of recovery and decreases the incidence of nausea, vomiting, and hypotension, without increasing the risk of bradycardia or delaying extubation (30). Nevertheless, the optimal OFA protocol for enhancing recovery quality remains to be clarified. Liu et al. reported that an OFA TIVA strategy consisting of esketamine, dexmedetomidine, lidocaine and a cervical plexus block improved postoperative quality of recovery, reduced PONV, and lowered pain scores in thyroid surgery (31). Similarly, another randomized clinical study indicated that an OFA TIVA strategy with esketamine, dexmedetomidine, and lidocaine significantly decreased PONV in lower extremity wound surgery (32). By contrast, a recent randomized controlled trial in gynecological laparoscopic surgery found that OFA with sevoflurane did not significantly reduce PONV or pain (3). However, this study employed a higher concentration of sevoflurane in the OFA group compared with the OBA group. As inhalation anesthesia is associated with an increased incidence of PONV relative to TIVA with propofol, this methodological choice may have attenuated the potential benefits of OFA.

In the present study, we have refined the OFA protocol by integrating regional nerve blockade with TIVA to optimize both analgesia and quality of recovery. Specifically, patients in the OFA regimen will receive dexmedetomidine infusion (0.6 μg/kg over 10 min at induction, followed by maintenance infusion of 0.2–0.7 μg/kg/h), propofol (2–2.5 mg/kg for induction, followed by a continuous infusion of 50–150 μg/kg/min), esketamine (0.3 mg/kg for induction, with supplemental boluses of 0.1–0.2 mg/kg as required), and intravenous lidocaine (1 mg/kg). Following induction, bilateral TAP block will be administered. To minimize PONV risk, all patients will undergo propofol-based TIVA together with prophylactic dexamethasone and ondansetron. A notable strength of our study is that it represents the first multicenter randomized controlled trial to evaluate the effects of OFA on quality of recovery in gynecological laparoscopic surgery.

This study also has limitations. First, some patients will be discharged within 72 h after surgery, and their quality of recovery will be assessed only via telephone follow-up. Second, dexmedetomidine infusion may induce bradycardia, potentially providing subtle cues that could compromise blinding. Third, the optimal strategy for administering OFA in gynecological laparoscopic surgery remains uncertain. Our protocol is derived from a limited number of prior studies and thus requires further validation. Fourth, the narrow BMI range and the exclusion of patients with significant comorbidities may limit the generalizability of the study protocol and its findings.

In conclusion, this multicenter randomized controlled trial will determine whether an intraoperative intravenous OFA regimen improves the quality of recovery relative to a traditional intraoperative OBA technique in women undergoing gynecological laparoscopic surgery.
