# Supplementary material for: Effect of total intravenous opioid-free anesthesia on quality of recovery following gynecological laparoscopy: protocol for a multicenter, randomized, double-blind, controlled trial
Source: Front Med (Lausanne). 2026 Feb 9;13:1747328. doi: 10.3389/fmed.2026.1747328 (PMC12926402; doi:10.3389/fmed.2026.1747328)
Supplement: Supplementary file 2 [file Data_Sheet_2.docx]

**Table S1. Definitions of adverse events within 72 hours after surgery according to the CTCAE─Version 5.0**

| **Event** | **Definition** |
| --- | --- |
| Hypotension | Mean blood pressure (MBP) decrease of > 30% from baseline or MBP < 65 mmHg. |
| Bradycardia | Heart rate (HR) < 50 beats/min. |
| Hypertension | MBP increase of > 30%. |
| Tachycardia | HR > 100 beats/min. |
| Oversedation | Ramsay Sedation Scale score 5–6 (1 = anxious/agitated; 2 = cooperative/oriented/tranquil; 3 = drowsy but responsive to commands; 4 = asleep with brisk response to light glabellar tap or loud auditory stimulus; 5 = asleep with sluggish response to noxious stimuli; 6 = unarousable). |
| Desaturation | peripheral oxygen saturation (SpO2) < 90% after extubation. |
| Dizziness | A common functional cerebral disorder. Feeling dizzy within 72 hours after surgery. |
| Headache | Feeling intermittent or persistent headache within 72 hours after surgery. |
| Ileus | Obstruction of passage of intestinal contents from any cause. Diagnosis can be based on the four major symptoms of abdominal pain, vomiting, abdominal distension, and cessation of defecation. Visible bowel patterns or peristalsis in the abdomen waves and hyperactive bowel sounds are also typical clinical symptoms. |
| Hyperalgesia | Pain elicited by non-noxious tactile stimuli or exaggerated pain response to minor stimuli. |
| Hallucinations | A Psychiatric disorder characterized by a false sensory perception in the absence of an external stimulus. |
| Agitation | A Psychiatric disorder characterized by a state of restlessness associated with unpleasant feelings of irritability and tension. |
| Nightmares | A distressing or extremely disturbing dream. |
| Delirium | A Psychiatric disorder characterized by the acute and sudden development of confusion, illusions, movement changes, inattentiveness, agitation, and hallucinations. Usually, it is a reversible condition. |

CTCAE, common terminology criteria for adverse events; HR, heart rate; MBP, mean blood pressure; SpO2, oxygen saturation.
